# Time Pressure Affects the Risk Preference and Outcome Evaluation

**DOI:** 10.3390/ijerph20043205

**Published:** 2023-02-11

**Authors:** Chiuhsiang Joe Lin, Huiqiao Jia

**Affiliations:** 1 Department of Industrial Management, National Taiwan University of Science and Technology, Taipei 106335, Taiwan; 2 Institute of Human Factors & Safety Engineering, Hunan Institute of Technology, Hengyang 421002, China

**Keywords:** time pressure, decision-making, risk preference, outcome evaluation, FRN

## Abstract

It is ubiquitous that food delivery riders do not have unlimited periods of time for deliberation to make decisions. Time pressure plays a significant role in decision-making processes. This study investigated how time pressure affected risk preference and outcome evaluation through behavioral and electrophysiological responses during decision-making. Participants finished a simple gambling task under three different time constraint conditions (high/medium/low). Behavioral and event-related potentials (ERPs) data were recorded during the experiment. The results showed that the decision time of people was shorter under high time pressure than under medium and low time pressures. People tend to make more risky choices when under high time pressure. The feedback-related negativity (FRN) amplitude was smaller in the high time pressure than in medium and low time pressure conditions. These findings provided evidence that time pressure has an impact on the risk decision-making process.

## 1. Introduction

Recently, the significant accounts of crashes and risky behaviors among delivery riders have been a serious concern for the public. Since the COVID-19 pandemic, people are increasingly ordering food and daily necessities through delivery platforms, which expands the demand for delivery riders. According to the Meituan Research Institute, the number of food delivery riders signed up to Meituan platforms reached 2.952 million in the first half of 2020, an increase of 415,000 compared with the first half of 2019 [1]. By the end of 2021, the number of food delivery riders in China reached 13 million, nearly 1% of the country’s population (National Bureau of Statistics, Beijing, China) [2].

However, more and more risky behaviors were undertaken by food delivery riders, ranging from violating traffic regulations (driving the wrong way, running red lights, and selecting dangerous travel speeds) to hit-and-run accidents that endanger people’s lives. Although a large number of accidents have emerged as a painful lesson that there may be danger/loss in the situation, many delivery riders still choose risky behaviors. One important factor leading to the growing number of delivery riders’ dangerous behaviors and accidents is the strict time limits from the delivery platform [3]. Therefore, it is worth discussing why people engage in risky behaviors under time pressure caused by time limitations. Understanding how time pressure affects choices under risk is an important topic from both a practical and a theoretical standpoint.

As one of the major topics in the human judgment and decision field, risk decision-making could be affected by time pressure. When the duration of the decision process is limited by external factors (e.g., deadline or time constraints), the time available for making choices definitely has an impact on both the information processing and the quality of the final decision [4]. According to earlier studies, time pressure can affect the risk tolerance of people, described as making people more risk-averse or less risk-averse [5,6]. Although the observations seem to be the opposite, such an effect would have significant consequences since it suggests that risk decisions are systematically distorted when under time pressure.

Outcome evaluation is a critical stage of decision-making, which can be used to assist people to make decisions and modify their decision strategies [7]. As is known, risk decision-making is based on complicated cognitive manipulations, including subjective perception and cognition of risks, the sensitivity of gains and losses [8], and previous experiences [9]. As a result, the processing of outcome feedback could be a crucial factor that might have an impact on risk decision-making. According to a study, the differences between individuals in risk-taking behaviors are frequently attributed to differences in how risks and rewards are perceived and evaluated [10]. Since the ability to process feedback information is crucial in risk decision-making, the feedback outcome evaluation may influence people’s choices. For delivery riders, risky behaviors could lead to different outcomes. The choice of risky behavior may bring two kinds of results. The positive outcomes could be on-time delivery or early delivery, favorable reviews, and more chances to line up orders from the delivery platform. The negative outcomes might be penalties or traffic accidents. On the contrary, if the delivery rider makes risk-averse choices, the positive outcome is still the on-time delivery, while the negative outcome may be a timeout for delivery but staying safe. However, how time pressure affects people’s evaluation of decision outcomes is not fully understood, especially the cognitive processing and brain activities when people face choices where the outcomes are uncertain and unpredictable in advance.

Event-related potentials (ERPs) have been commonly used to study the cognitive processes of the brain. Compared to other neurophysiological measures, ERPs are characterized by non-invasiveness, high temporal resolution, and low cost [11]. With the advantage of high time resolution, ERPs are well suited to explore the dynamic mechanisms of cognitive processes, especially the stage of outcome evaluation in decision-making [12]. The feedback-related potentials (FRN) are a fronto-central ERP component elicited between 200 and 300 ms following the feedback onset that has been localized to the anterior cingulate cortex (ACC) [13] and by far is the most extensively investigated feedback processing ERP component [14]. FRN is sensitive to the processing of outcome evaluation [15] and is often used to examine cognitive processes correlated with risky decision-making and feedback evaluation processing [9,16]. Furthermore, the FRN component represents an early, rapid stage of outcome processing [17] and represents the brain mechanisms that distinguish between favorable and negative outcomes [18]. Additionally, FRN is reported to respond more strongly to negative situations (e.g., financial losses; erroneous feedback) than positive situations [19,20].

As mentioned above, time pressure could impact risky decision-making. Meanwhile, the specific underlying mechanisms through which time pressure exerts effects on risky decision-making remain unclear. Although some researchers began to focus on the cognitive processing undertaken when risky decision-making in neuroscience [21], the work was very limited and the neuro-mechanism especially the outcome processing is still not fully understood. To our knowledge, no ERP studies have been conducted that directly compare the electrophysiological reactions of feedback processing under different time constraints. Therefore, we used a monetary gambling task to investigate how time pressure affects both the behavioral performance and electrophysiological activity of outcome feedback processing.

## 2. Literature Review

As an advanced cognitive activity, decision-making is a process of evaluating and selecting alternatives, and the behavior that humans employ to meet the requirements of the environment. Discovering the factors that may affect decision-making is critical for humans, for this ability plays an important role not only in environmental adaption and survival but also in safety-critical performance in many important jobs. Decision-making always requires the ability to choose between competing options that will produce diverse outcomes [22]. As a major type of decision-making involving uncertainty, risky decision-making is counted among the most active research topics [9].

The relationship between time pressure and human behaviors has been brought into focus for many years. When people have to make a judgment or decision in constrained time, time pressure is experienced. As the time pressure increases, the judgment or decision may change for the following reasons: (1) the lack of time to deliberate the choice options, (2) applying different cognitive strategies, or (3) focusing on different aspects of the choices (e.g., outcomes). This is a crucial problem, especially in the risky decision-making domain.

Time pressure has been found to impact cognitive performance during decision-making in various ways. Decision-making requires the synergistic effects of various cognitive skills [23]. Studies on the cognitive processing involved in making decisions have shown that high time pressure can induce perceptual narrowing, which decreases vigilance, working memory, and the utilization of available clues [24,25,26,27]. In addition, people may not have enough time or the attentional resources required to review and weigh a variety of potential hypotheses under time pressure in fast-paced event-driven situations [25,28]. Moreover, time pressure has been observed to lead to more rigid behavior, manifested as the failure to alter and adapt behavior to a new situation [29]. This is due to the reduced information processing and the constriction in the control system caused by time pressure. However, the effect of time pressure on decision-making is not necessarily to be negative. Time pressure may also lead to faster processing of information and shorter response time. According to findings by Payne et al., participants under moderate time constraints accelerated their processing and tried to work faster without changing their processing patterns [30].

Time pressure could influence risk preference/risky choice behavior. Early research showed that the weighting of positive and negative information can be affected by time pressure, by shifting judgments toward already-known information rather than externally delivered information [31]. In one study, people tended to put more weight on negative information and less on positive information as the time pressure increased in a consumer choice task [32]. It implied that time pressure would enhance reliance on negative information in decision-making processes. Another study has demonstrated a similar emphasis on negative information when deciding on a risky financial gambling task. People are more risk-averse under high time pressure, compared to medium and low time pressure; people tended to be sensitive to the negative outcome under high time pressure, whereas sensitive to the positive outcome under lower time pressure [6]. However, there are some different observations. Goldstein and Busemeyer studied performance in a gambling task and found more risky gambles were chosen under high time constraints. People’s preferences were reversed as the time pressure reduced so that the less risky gamble became the more frequent choice [33]. Dror’s study observed that under time pressure, participants were more conservative at the lower risk levels but were more prone to take risks at the higher levels of risk [34]. Madan et al. found that time pressure increases risk seeking in decisions [5], and Olschewski et al. found a similar tendency that risk preferences under time pressure are not in accordance with the no time pressure situation [35]. Although these findings seem to be different, it implies that time pressure could distort the choices during risky decision-making.

Although there are many studies that have observed the influence of time pressure on decision-making, the findings are somewhat mixed. Therefore, clarifying the relationship between time pressure and risky decision-making remains a significant topic.

## 3. Methods

### 3.1. Participants

Thirty-eight undergraduate students (14 females and 24 males, mean age 20.37 ± 1.55) from the Hunan Institute of Technology participated in the experiment, and they all received course credit for their participation. In addition to having the normal or corrected-to-normal vision and no known neurological disorders, all subjects were right-handed. None of the participants have ever participated in a similar experiment. The Human Institute of Technology’s Research Ethics Board gave its approval for this study, which was carried out in conformity with the Declaration of Helsinki. To participate in the study, each subject signed an informed consent.

### 3.2. Experimental Stimuli and Procedures

**Figure 1:**
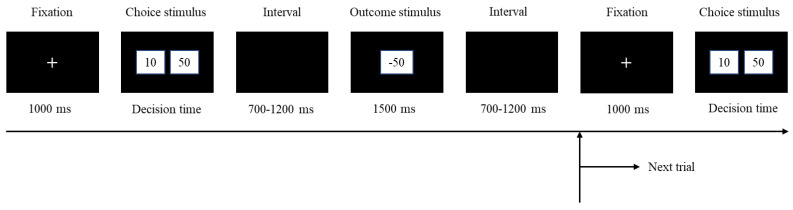
Single outcome gambling task.

A single outcome gambling task programmed with E-prime 2.0 was used to set the decision-making situation and outcome feedback. During the task, participants were required to choose between risky binary gambles (two boxes labeled “10” or “50”, each representing a monetary value) [36]. Feedback is given following the decision to indicate whether the participant gained or lost, along with the value of either outcome (see Figure 1). The box labeled “50” is the higher-valued choice, and choosing it is regarded as risky. Every participant needed to complete 3 blocks of 192 trials. The blocks were designed for different time constraints. According to previous research [37], the choice options were respectively presented for 1000 ms, 2000 ms, and 3000 ms in different blocks, which represent high time pressure, medium time pressure, and low time pressure. The blocks were randomly arranged. The probabilities of loss or gain feedback in each block were equal (50%), which was unbeknownst to the participants. The order of loss or gain feedback was also pseudo-randomized.

Before the formal experiment, all participants were informed of the monetary gambling task, instructions for the task, and the ERP procedures. The goal for participants was to maximize their financial gain, or in other words, to earn as much money as possible. To increase motivation, participants were told that they would receive the money they earned in the tasks at the end of the experiments.

In a sound-isolated, darkly lit room, the participants were placed in comfortable chairs 100 cm in front of computers during the experiment. A total of 576 trials of the gambling task had to be finished by each participant. A fixation cross appeared in the center of the screen for 1000 milliseconds at the start of each trial of the gambling task. Then, two boxes (the left-hand box was labeled “10” while the right one was labeled “50”) appeared in the center of the screen. The numbers 10 and 50 represented a wager of 10 and 50 Jiao RMB, or about 20 and 80 US cents, respectively. The options stayed on the screen until one of the numbers was chosen by the participant pressing the F or J key on the keyboard with their left or right index finger (F for the alternative on the left and J for the one on the right). The outcome of the participant’s choice then showed up with a “+” or “−” symbol, denoting a gain or loss of 10 or 50 Jiao RMB, respectively. If the participant did not react during the response period, then the program would show a “Time Out” message in the middle of the screen. The feedback display remained visible for 1500 ms. Before the start of the following trial, there was a brief period (randomly varied between 700 and 1200 ms) during which a blank screen was displayed. The stimuli were shown and behavioral responses from the participant were gathered by the program.

### 3.3. EEG Recording and Analysis

Using an elastic cap and sintered Ag/AgCl electrodes, electroencephalogram (EEG) data were collected from 64 scalp sites following the Extended International System frontal lobes. Horizontal electrooculogram (HEOG) data were recorded from electrodes placed at the outer canthi of both eyes. Vertical electrooculogram (VEOG) data were recorded from electrodes placed above and below the left eye. The impedance of each electrode was kept below 5 kΩ. With a bandpass filter of 0.01 to 100 Hz, EEG and EOG signals were sampled at 1000 Hz continuously.

The EEGlab toolbox of MATLAB R2021b (MathWorks, Natick, MA, USA) was used to preprocess the data. The preprocessing steps were data integration, electrodes’ location, re-reference setting, data filtering, segmentation, baseline correction, EOG removal, artifacts rejection, etc. A finite impulse response filter with a bandwidth of 0.01–30 Hz and zero phase distortion were used to filter the collected EEG data. The segmentation of the filtered data started 200 ms before the outcome and lasted for 1000 ms. Following baseline correction for the mean voltage throughout the 200 ms prior to the onset of the outcome for each epoch, the association with four separate feedback conditions—gain10, lose10, gain50, and lose50—was averaged.

FRN was picked for investigation in this study. ERPs were averaged on the basis of the EEG data. According to earlier studies, FRN is maximal in the fronto-central region [20]. We concentrated on FRN at Fz, Cz, and Pz electrodes since prior studies [19,20] revealed that these components were the largest in these electrodes. The FRN amplitude was calculated as the mean amplitude within a temporal window of 200–350 ms, based on previous research and visual inspection. 

### 3.4. Statistical Analysis

We conducted separate analysis of variance (ANOVA) models to examine the effects of time pressure on behavioral reactions and brain activities during risky decision-making. To investigate whether the time pressure influenced the behavioral reaction during risky decision-making, decision times and risk ratios of participants were examined using repeated measures analysis of variance (RMANOVA), with time pressure treated as the within-subjects variable. To investigate whether the time pressure affected the brain activities during risky decision-making, the amplitude and latency of the FRN were analyzed using RMANOVA with time pressure, outcome valence, and outcome magnitude treated as the within-subjects variables.

SPSS Statistics 25.0 (IBM, Somers, New York, NY, USA) was used to conduct the statistical analysis. The significance level was set at 0.05 for each of the analyses included in this investigation. If the sphericity assumption of the ANOVAs was violated, the Greenhouse—Geisser correction was applied to adjust the degree of freedom and the *p* values [38]. Post-hoc testing of significant effects was conducted with the Bonferroni method to address the Type I error inflation [39]. Simple effects models were used to assess significant interactions in the ANOVAs. The effect size in the ANOVA testing was shown using partial eta-squared (η^2^p), where 0.05 indicated a minor effect, 0.10 represented a medium effect, and 0.20 denoted a high effect [40].

## 4. Results

The behavioral reaction of the participants was analyzed using repeated measures ANOVA. Table 1 shows the behavioral results under three different time pressures.

### 4.1. Behavioral Results

#### 4.1.1. Decision Time

**Figure 2:**
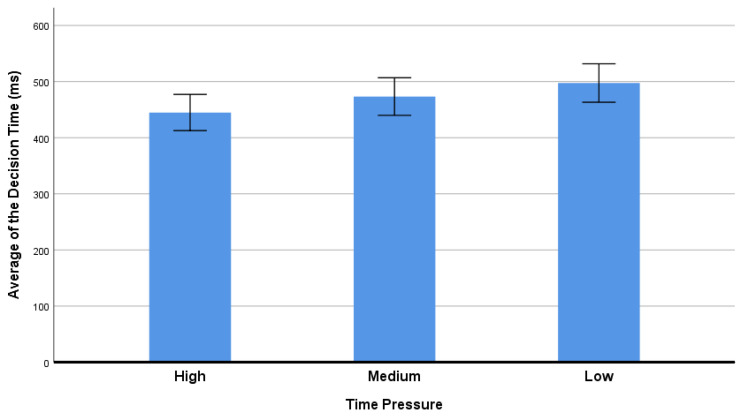
The average decision time of different time pressures. Error bar = 95% confidence interval.

As shown in Table 1 and Figure 2, the average decision time of the participants was significantly different under the three levels of time constraints [F (1,2) = 44.529, *p* < 0.001, η^2^ = 0.546]. A post hoc pairwise comparison test showed that the average decision time was lower in the high constraint condition than in the medium and low time constraint conditions. In addition, the decision time is higher in the medium than that in the low time constraint condition.

#### 4.1.2. Risky Choices

In the gambling task, option “50” was defined as the risky choice (big potential gain or loss), and option “10” was the risk-avoidant choice (small potential gain or loss). The propensity to select the riskier choice shows a preference for a risky strategy. By dividing the number of risky options by the total number of options, this preference was calculated as the “risky ratio.” It was unnecessary to calculate the “risk-avoidant ratio,” because the value of the risk-avoidant ratio was equal to one minus the risky ratio in each condition. Since the “risk-avoidant ratio” in each scenario was equal to one minus the “risky ratio,” the calculation of the “risk-avoidant ratio” was not necessary.

**Figure 3:**
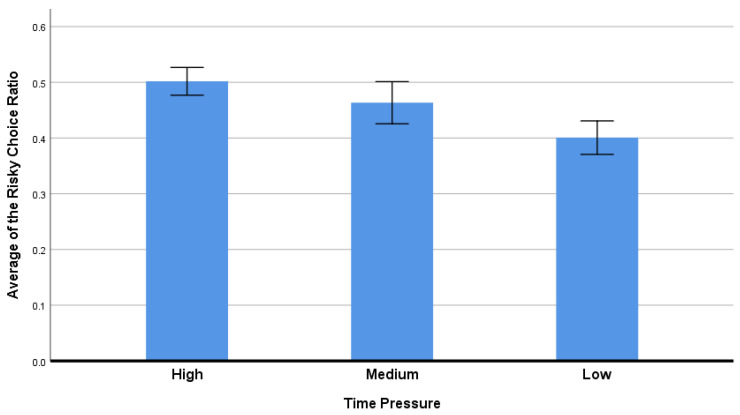
The risky ratio of different time pressures. Error bar = 95% confidence interval.

The ratio of risky decisions made by each participant under the high, medium, and low time constraints were further examined using repeated measures ANOVA. The RMANOVA results showed that there was a significant difference among the three different time constraint conditions [F (1,2) = 20.682, *p* < 0.001, η^2^ = 0.359]. A post hoc pairwise comparison test showed that the average ratio of risky choices was higher in the high constraint condition than in the medium and low constraint conditions. In addition, the ratio of risky choices is higher in the medium than in the low time constraint condition (See Figure 3). 

### 4.2. EEG Results

**Figure 4:**
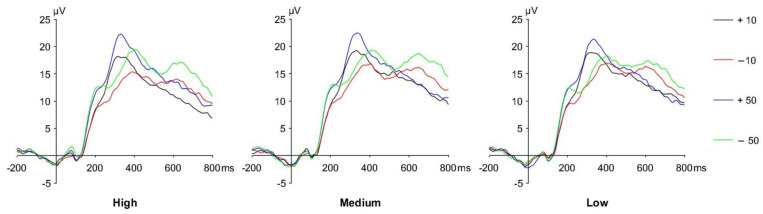
The grand average ERP waveforms of different time constraint conditions.

The grand average ERP waveforms induced by 3 different time constraint conditions (High, Medium, Low) and 4 different outcome stimuli (+10, −10, +50, −50) are depicted in Figure 4.

**Figure 5:**
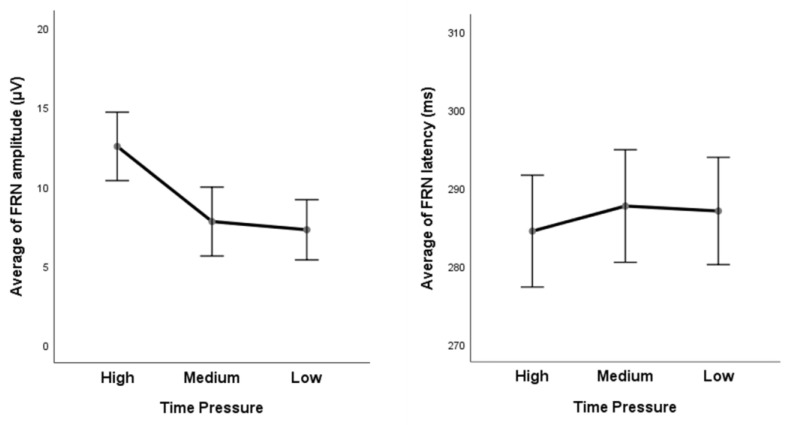
The average FRN amplitude (**left**) and latency (**right**) of different time pressures. Error bar = 95% confidence interval.

**Figure 6:**
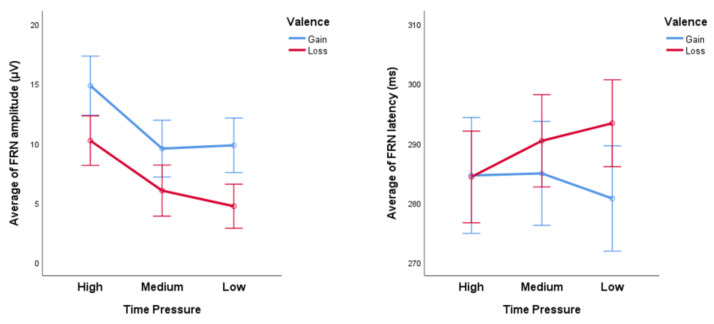
The average FRN amplitude (**left**) and latency (**right**) of different valences under different time pressures. Error bar = 95% confidence interval.

**Figure 7:**
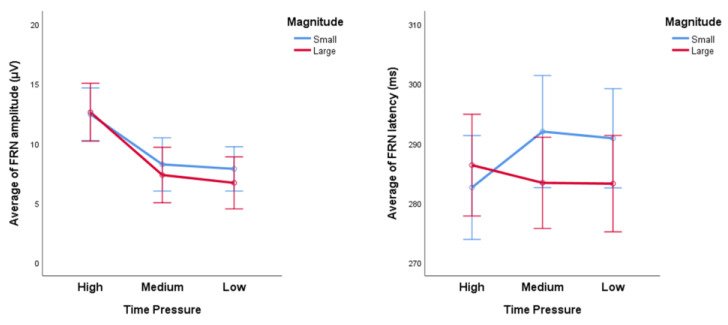
The average FRN amplitude (**left**) and latency (**right**) of different magnitudes under different time pressures. Error bar = 95% confidence interval.

The average FRN amplitude and latency under different time pressures are shown in Figure 5. The average FRN amplitudes of high, medium, and low time constraint conditions are, respectively, (12.533 ± 1.064) μV, (7.793 ± 1.072) μV, and (7.275 ± 0.937) μV. The average FRN latency of high, medium, and low time constraint conditions are, respectively, (284.480 ± 3.530) ms, (287.684 ± 3.553) ms, and (287.053 ± 3.387) ms. The average FRN of Time Pressure × Valence and Time Pressure × Magnitude are, respectively, shown in Figure 6 and Figure 7.

Results from the repeated measures ANOVA are reported in Table 2. The analysis of FRN amplitudes revealed significant effects of time constraint difference [F (1, 2) = 41.329, *p* < 0.001, η^2^ = 0.527] and feedback valence [F (1, 2) = 75.593, *p* < 0.001, η^2^ = 0.671]. Pairwise comparison showed that FRN amplitudes of high time constraint conditions are significantly less negative than medium and low time constraint conditions, while the FRN amplitude in the medium time constraint condition is significantly less negative than that in low time constraint conditions. FRN amplitudes of “loss” are larger than “gain” [(6.991 ± 0.896) μV vs. (11.410 ± 1.078) μV]. Considering FRN is a relatively negative component, it should be noted that “larger FRN” indicates a more negative component.

Additionally, a main effect of the feedback valence was found in the analysis of the FRN latency. The FRN latency of loss condition is longer than the gain condition [(289.382 ± 3.140) ms vs. (283.430 ± 3.456) ms, F = 4.326, *p* = 0.045 < 0.05, η^2^ = 0.105]. No further significant effects or interactions were found from the RMANOVAs.

## 5. Discussion

Time pressure has been demonstrated to influence decision-making in various ways, but the results have been somewhat mixed (as mentioned in the introduction). The motivation for the current experiment came from these contradictory results as well as the lack of research that examined the brain’s processes during decision-making. We explored the behavior and brain responses when people were faced with risky choices and different outcome feedback to broaden the applicability of time pressure findings.

The current study used both behavioral and electrophysiological measures to evaluate the association between time pressure and decision-making in a simple monetary gambling task. The impact of time pressure on decision-making was shown using both behavioral and ERP results, although they highlighted different distinct facets of this influence.

### 5.1. The Effect of Time Pressure on Behavioral Performance

The average decision time was significantly different under the three different time constraint conditions in the current study. Participants took a shorter time to make decisions in the high time-constraint condition than in medium and low time conditions. Although some researchers claimed that in response to time pressure, people might have some difficulties in cognitive processing [41,42], it does not mean that people necessarily need more time to make decisions. In addition, in many real-life situations, people do not always have enough time as well as the cognitive resources to make decisions. For instance, to prevent an accident, air traffic controllers must make quick decisions when managing aviation traffic. Drivers must decide whether to accelerate through the intersection at a high rate of speed or slam the brakes on to stop before the red light appears after seeing an immediate yellow light signal. Police officers must decide rapidly regarding when to use force [43]. Furthermore, moderate time pressure could lead to faster processing of information and shorter response time during decision-making [21,44,45]. In the current study, we found a similar phenomenon—with higher time pressure, people tended to make decisions more quickly. The results indicated that participants could react and make decisions more quickly with some time pressure. 

Compared to the medium and low time constraint conditions, participants tended to select option 50 (high risk and high return) more frequently under the high time constraint condition, suggesting that people preferred to make riskier decisions when facing high time pressure. The finding was in line with Young’s earlier research, which suggested that time pressure increased people’s tendency to risk-seek during decision-making [45]. Many previous studies also support that time pressure encourages people to be more risk-seeking and to make more riskier decisions [5,35,46,47].

On the contrary, there are some different opinions. Several researchers discovered that people in their studies become more risk-averse while under time pressure, for the reason that more weight is given to negative information as time pressure increases [47]. Ben Zur and Breznitz claimed that, compared to medium and low time pressure, people with high time pressure tended to make more safe choices. The explanation for this statement was that participants tended to pay more time to the probability of negative outcomes [6]. According to these researchers, time pressure is a stressful condition that could increase the apprehension induced by the threat of negative consequences; a less risky choice might be an available solution for lowering such apprehension, contributing to feelings of safety while under stress.

It is noteworthy that there are some differences between Ben Zur and Breznitz’s study and the current study. Firstly, the time pressure setting was different. In Ben Zur and Breznitz’s study, the time pressure conditions were set as High (8 s), Medium (16 s), and Low (32 s). In the current study, we set the time constraint as High (1000 ms), Medium (2000 ms), and Low (3000 ms), which were stricter and in accordance with Busemeyer’s study [37] to make sure the time pressure was induced successfully. Secondly, the task set was different. Participants were individually asked to finish 3 blocks, which totaled 36 trials of gambles in their study. In contrast, a simple gambling task was used as the main task which consisted of 3 blocks and totaled 576 trials in the current study. Although the risk decision task (gambling task) seems to be similar between the two studies, the different settings could be one factor that leads to different results.

### 5.2. The Effect of Time Pressure on Brain Activities

Much research has proved that decision-making could be influenced by time pressure. People often require feedback information from the environment to determine the success of their decision, and thus, this affects their future decisions. However, whether the processing of outcome feedback could be affected by time pressure is still not clear. In this study, the FRN amplitude was found to be significantly different under different time pressures. The FRN amplitude was less negative (smaller) in the high time constraint condition than that in the medium time constraint condition, and less negative in the medium time constraint condition than that in the low time constraint condition.

Numerous earlier studies have shown that the FRN component is directly associated with risk-taking while making decisions [13,48,49]. In addition, risk-seeking individuals showed a lower FRN amplitude than risk-averse individuals [50]. These indicated that the increase in FRN amplitude could be an index for risk-seeking. It is also coincident with the risky choice ratio as shown in the behavioral results. Under high time pressure, people tend to make more risky choices than medium and low time pressures. Meanwhile, the FRN amplitude is smaller under high time pressure than under medium and low time pressures.

Another explanation for these findings may be the notion that the FRN amplitude increases with perceived control over outcomes [14]. Additionally, larger FRN amplitudes suggest that the current event has a stronger motivational impact [13]. The more time pressure, the fewer opportunities people have to make a deliberate decision for the option they prefer, hence the lower the perceived control over outcomes. A decrease in overall FRN amplitude under time pressure may hence reflect the notion that people have less control in obtaining preferred outcomes, as they are given less opportunity to deliberate about preferred outcomes. This reduced agency may also explain why people tended to make more risky decisions under time pressure. The natural tendency to make risk-averse choices (prospect theory by Kahnemann and Tversky) [8] may be reduced if there is less opportunity to make deliberate choices, leading to more arbitrary choices (i.e., 50—50, as seen in this study). Indeed, decisions underlie a strong speed—accuracy trade-off [51]. From this perspective, a stricter response deadline is expected to induce faster, but less “deliberate” decisions. This may also be why delivery riders are more likely to take risks when they are under time pressure.

In general, although many researchers have studied a similar topic to the current study, the research paradigms are much different. The simple outcome gambling task we adopted in this study is widely used in exploring risky decision-making, especially the outcome evaluation [9,13,19]. Compared with previous studies that paid more attention to individuals’ performance and got mixed observations, this study examined the effects of time pressure on risky decision-making using both behavioral and electrophysiological methods. In addition, some research began to focus on brain activities under time pressure [21,44], but the decision-making process and the outcome evaluation have not been discussed. Neuropsychological analyses help in finding interconnections between time pressure and outcome information processing and the neuro-mechanism of decision-making under time pressure, which further verifies the influence of time pressure on risky decision-making.

### 5.3. Limitations and Future Research

Some limitations must be considered in interpreting the findings. Due to the COVID-19 pandemic, it is not possible to invite food delivery riders to participate in the experiment. Furthermore, the gambling task under time pressure might not show the food delivery riders’ real situation. However, the findings still have implications for the practice and could provide evidence for the necessity to improve the time pressure from delivery platforms. Future studies should investigate the behaviors of real food delivery riders in a situation that is closer to reality.

Another issue needing clarification is the human conditions in this study. People’s performance and cognitive activities could easily shift under different mental and physical states. To avoid the impact of human conditions, all the participants in this study were rested and provided with a clear view. Therefore, the findings of the current study may be well applicable to the optimal condition only; however, other concurrent factors may exist during real work environment. Future studies could consider other human factors such as mental and physical states during work.

In summary, the current study investigated whether time pressure impacts people’s decision-making and outcome evaluation by analyzing behavioral responses and brainwave parameters. Indeed, findings in the present study revealed that time pressure has an impact on both the decision behaviors and the FRN component. People tend to make quick and risky choices under high time pressure. The results of the ERP suggest that this phenomenon may be attributed to time pressure that interferes with decision-making, particularly with the processing of outcomes. The implication of the findings is that controlling the time constraint for people who might be facing risky choices is necessary. This study cannot only outline from the neurophysiological level how time pressure can explain people’s risk decisions, but it also provides a basis for the irrationality of delivery platforms setting time limits.

## 6. Conclusions and Implications

The current study illustrates how time pressure affects risky decision-making. Under high time pressure, people tend to be more risk-seeking. By demonstrating that time pressure has an impact on both risk preferences and outcome evaluation, the present study advances our understanding of how time pressure influences decision-making. These findings shed light on the electrophysiological processing involved in risky decision-making and have important implication for making appropriate decisions when decision makers are faced with time pressures.

In addition, the findings in this study could provide some positive implications for practice. First, since people’s tendency to make more risky choices under high time pressure, managers should be more cautious about the setting of time limits or deadlines for a specific work or task. Second, as the brain activities showed the differences under different time pressures, it provides a possibility for wearable devices to be used for dynamically monitoring the decision process of operators or decision-makers. If the decisions seem to be riskier or may lead to danger, an alert could be given to the decision-makers. These implications can be applied not only to the work of food delivery riders, but also to other types of jobs or tasks involving risky decision-making.

## Figures and Tables

**Table 1:** Behavioral results.

	Time Pressure				
	High	Medium	Low	F	*p*
Decision time (ms)	444.840 ± 15.924	473.314 ± 16.526	497.462 ± 16.905	44.529	0.000 ***
Risky Choice Ratio (%)	0.502 ± 0.012	0.463 ± 0.019	0.401 ± 0.015	20.682	0.000 ***

*** *p* < 0.001 indicates statistically significant differences.

**Table 2:** Repeated measures ANOVA results.

Variables or Interactions	FRN			
	Amplitude		Latency	
	F	*p*	F	*p*
TP	41.239	0.000 ***	0.576	0.564
V	75.593	0.000 ***	4.326	0.045 *
M	1.754	0.194	1.734	0.196
TP × V	1.433	0.245	2.424	0.096
TP × M	0.989	0.377	2.801	0.067
V × M	0.578	0.452	1.817	0.186
TP × V × M	0.574	0.566	2.725	0.072

TP represents Time Pressure, V represents Valence, and M is for Magnitude. * *p* < 0.05 and *** *p* < 0.001 indicate statistically significant differences.

## Data Availability

The data presented in this study are available on request from the corresponding author.
